# 

**Published:** 1875-11

**Authors:** 


### RENEWAL OF SUBSCRIPTIONS.

Our subscribers should not forget that thousands of subscriptions expire with the December number, and that such should renew at once, in order that we may know what number to print in January. We hope all will renew, and that such friends as have leisure and influence, will encourage others to become regular readers of our journal. We hope to make our magazine far better the coming year than it has ever been.

---

THE NELATON SUPPOSITORY, for piles and constipation, introduced by Hall & Ruckle, 218 Greenwich St., is really a capital thing for all cases of the kind alluded to. It is not a medicine, but a local application, which, while lubricating the inflamed parts, relieves the soreness and pain, reduces the inflammation, and ultimately heals all fissures and abrasions. The sense of comfort is almost instantaneous. We are using it every day among our patients.

